# Infant Feeding Practices and Their Association with Early-Life Nutrient Intake: Baseline Findings from the Baby-Act Trial

**DOI:** 10.3390/dietetics4020015

**Published:** 2025-04-04

**Authors:** Cristina Palacios, Elvira Alvarez, Maria Gabriela Kallis, Yari Valle, Jeremy Pomeroy, Maribel Campos

**Affiliations:** 1 Department of Dietetics and Nutrition, Robert Stempel College of Public Health & Social Work, Florida International University, Miami, FL 33199, USA;; 2 Center for Community Outreach for Health Across the Lifespan (COHeAL), School of Dental Medicine, Medical Sciences Campus, University of Puerto Rico, San Juan P.O. Box 365067, Puerto Rico;; 3 Fritz Wenzel Center for Clinical Research, Marshfield, WI 54449, USA;

**Keywords:** infant, feeding, diet, Puerto Rico, WIC

## Abstract

**Introduction:**

This is a secondary cross-sectional analysis of participants’ baseline data from the Baby-Act Trial to compare energy and macronutrients intake by feeding type. This was a cluster-randomized clinical trial among pregnant women and their infants participating in the Puerto Rico WIC program to test the clinical effectiveness of an intervention that addressed various infant obesity risk factors during the first year of life.

**Methods:**

Participants completed at baseline a sociodemographic questionnaire and a validated infant food frequency questionnaire (FFQ). The FFQ was analyzed for type of feeding (exclusively fed breastmilk, fed breastmilk and formula, or exclusively fed infant formula) and for energy and macronutrient intake (protein, carbohydrate, and fat). Analysis of covariance was used to compare intake type of feeding, adjusting for maternal age. race, education, number of previous children, gestational length, and pre-pregnancy BMI.

**Results:**

The present analysis included 368 mother–infant pairs. Mean age of mothers was 26.7 years and of infants 0.7 months. Ten infants fed complementary foods were removed from the analyses. A total of 39.9% of infants were exclusively breastmilk-fed, 47.2% were fed breastmilk and infant formula, and 12.8% were exclusively formula-fed (n = 358). Intake of energy, protein, fat, and carbohydrates was significantly lower in infants fed only breastmilk compared to infants fed a combination of breastmilk and infant formula, and intake of protein and carbohydrates were lower in infants fed a combination of breastmilk and infant formula compared to infants fed only infant formula, after adjusting for important confounders.

**Conclusions:**

Very early infant feeding practices are associated with differences in dietary intake. The long-term health implications of this findings need to be further studied.

## 1. Introduction

Exclusive breastfeeding during the first six months of life is one of the key early exposures that the World Health Organization has recommended for healthy growth and development [1]. Several systematic reviews have shown that breastfeeding is associated with a lower risk of obesity later in life [2–4]. Differences in dietary intake between infants breastfed and infants formula-fed may be a contributory factor in this associations. In fact, several studies have consistently shown significantly lower intakes of energy and macronutrients among infants breastfed compared to infants formula-fed [5–8], and some have found a different growth trajectory among those breastfed [6–8]. For many years, studies have attributed these differences in growth trajectory to differences in protein intake among infants fed formula [9,10], but more recent studies have not found that a higher protein intake is associated with greater weight gain or higher weight velocity among healthy infants [11,12].

To our knowledge, none of these studies have included Hispanic infants, a group at a higher risk for excessive infant weight gain compared to Whites [13,14]. In particular, data from the Special Supplemental Nutrition Program for Women, Infants, and Children (WIC) in the US show that Hispanic children have one of the highest prevalences of obesity (17.4%) compared to non-Hispanic White (12.3%), non-Hispanic Black (11.9%), or Asian or Pacific Islander (10.7%) among those aged 2–4 years. Although data from WIC in Puerto Rico for 2020 revealed that 64.1% of infants were breastfed immediately after birth, only 31.4% of infants were exclusively breastfed for the first 3 months [15].

Therefore, the aim of the following analysis was to compare energy and macronutrient intake by feeding type (exclusively fed breastmilk, fed breastmilk and formula, exclusively fed infant formula) and by other infant and maternal characteristics at the baseline visit of participants in the Baby-Act Trial. This was a cluster-randomized clinical trial among pregnant women and their infants participating in the Puerto Rico WIC program to test the clinical effectiveness of an intervention that addressed various infant obesity risk factors during the first year of life [16].

## 2. Materials and Methods

### 2.1. Study Design

This is a cross-sectional secondary analysis of participants’ baseline data obtained from the Baby-Act Trial to compare energy and macronutrient intake by feeding type (exclusively fed breastmilk, fed breastmilk and formula, exclusively fed infant formula) and by other infant and maternal characteristics. The Baby-Act Trial is a cluster-randomized clinical trial implemented in collaboration with the Puerto Rico WIC program, to test the clinical effectiveness of an intervention that addressed various infant obesity risk factors among pregnant women and their infants during the first year of life [16]. Data collection started in June 2018, and all participants were recruited by April 2022. This analysis only includes the results from the baseline visit.

### 2.2. Setting

As previously described [16], this study was conducted in WIC clinics in Puerto Rico, a US territory in which 98.9% of the population is Hispanic. Participants were recruited from 16 clinics in the extended San Juan Metropolitan. Clinics were selected based on having enough space to conduct study-related activities (recruitment and assessments), having a relatively large number of pregnant participants to recruit from, and capacity to provide services following Hurricanes Irma and Maria in 2017 and the series of catastrophic events that ensued during the study implementation period. Study condition allocation was maintained as established before first study enrollment and confirmed via interim data analysis as established in the protocol’s analytical plan.

### 2.3. Subjects/Sample

Participants included healthy singleton pregnant women aged 18 years and older in the 3rd trimester who were participants of the WIC program and had access to the internet. Participants were excluded if they had high-risk pregnancies and/or with infants born before week 37 of gestation, with developmental disabilities, with severe conditions, or with prolonged hospitalization at birth (>7 days). Potential eligible participants were identified by the WIC staff at routine prenatal visits or in the clinic waiting room by our research staff. Eligibility criteria were assessed using a standardized screening form. The study was approved by the University of Puerto Rico–Medical Sciences Campus Institutional Review Board, and participants completed an informed consent before initiating study activities. Study engagement and fulfillment of data collection procedures transitioned to remote consent and data collection as Puerto Rico initiated a strict lockdown in May 2020 as part of the COVID-19 response plan. Approval for decentralized study procedures (remote informed consent process with electronic signature, remote data collection procedures and distribution of anthropometric assessment equipment for home collection) was achieved with the support of the Puerto Rico WIC program.

### 2.4. Assessments

The baseline visit was completed when infants were 0–2 months and included various questionnaires and measurements. We here describe the measures used in the present analysis.

- Socio-demographics: this questionnaire included maternal age, gender, race, ethnicity, educational level, number of children, duration of pregnancy, working status, use of prenatal vitamins and pre-pregnancy weight and height. Pre-pregnancy body mass index (BMI) was calculated as weight divided by height squared, and BMI was categorized as healthy weight (<25 kg/m2), overweight (25–29.9 kg/m^2^), and obesity (≥30 kg/m^2^). This questionnaire also asked about infant’s age, sex, ethnicity, and race.
- Diet: A validated infant food frequency questionnaire (FFQ) was used to assess diet [17]. This FFQ includes a list of 52 food items, with the method of preparation or description of the food. Participants were asked to recall the frequency that their infant consumed each food item in the last 7 days and the amount given using standard and commonly used serving sizes for this age group. The frequency of consumption of each food item was multiplied by the serving size to obtain the total food consumed per day, and it was reported in ounces per day. Based on the responses to the infant FFQ, the following calculations were performed:
  - Type of feeding: infants’ diets were categorized as: (1) Only breastmilk (if exclusively fed breastmilk); (2) Breastmilk and formula (if fed breastmilk and also formula); or (3) Only formula (if exclusively fed infant formula). Furthermore, infants’ diets were categorized as consuming or not complementary foods (foods other than milk).
  - Energy and macronutrient intake: this was calculated using a food database created using the Nutrition Data System for Research, developed by the Nutrition Coordinating Center of the University of Minnesota (version 25, 2014, program 2.8, Minneapolis, MN, USA) [18], as previously described and published [17]. This database was created for all the foods included in the FFQ based on foods typically consumed by infants in this age group [17]. For the infant formula, this database averaged the energy and nutrient values from the 10 most consumed infant formulas available in NDSR. To calculate the energy and nutrient for each food, the amount consumed per food per day was first calculated. For breastmilk volume intake, this was first calculated using the data from a recent meta-analysis of 167 studies conducted by Rios-Leyvraz and Yao [19] with breastmilk data for infants for each month from 0 to 24 months. The volume reported in Rios-Leyvraz and Yao [19] was used as the breastmilk volume by age in months, if infants were exclusively breastfed. If infants were partially breastfed, the volume in formula was subtracted from the volume as per that study [19], and what remained was considered as breastmilk volume intake. If the daily volume of formula exceeded the estimated total daily milk intake volume, a rule of 3 ounces (88.7 mL) per feeding was implemented, which was derived from the Feeding Infants and Toddlers Study (FITS) 2008 and 2016, the largest US cross-sectional surveys of caregivers of children from 0 to 48 months of age [20]. If infants were fed exclusively formula, the reported volume from formula was used as the total daily milk intake for this group. Once the volume of breastmilk and/or formula was established, as well as the amounts of other foods consumed by the infant per day, it was multiplied by the energy and macronutrient content of each food item per 1 ounce, as derived from the NDSR database directly. Lastly, the energy and nutrient amounts were summed for all food items to obtain total daily values. Estimated energy requirements were calculated per infant as per the Dietary References Intakes to determine if energy intake met these requirements [21].

### 2.5. Statistical Analysis

Descriptive statistics included mean and standard deviation or percentiles for quantitative variables and frequency for categorical data. To compare energy and macronutrient intake by feeding type, a multivariate analysis of covariance (MANCOVA) was used, adjusting for the following confounders: maternal age, race, education, number of previous children, gestational length, and pre-pregnancy BMI. Energy and macronutrient intakes were also compared by infant sex and maternal pre-pregnancy BMI categories (healthy weight, overweight, and obesity). All analyses were performed using SPSS Statistics software (version 28, IBM, New York, NY, USA).

## 3. Results

A total of 529 mothers were enrolled in the study; however, 161 participants either missed the anthropometric measures at baseline and/or did not complete the infant FFQ. Participants that did not complete the infant FFQ had similar socio-demographic characteristics than those that completed these measures, except that they had less children (0.61 vs. 0.77; *p* < 0.01). Socio-demographic characteristics of mothers and infants are shown in Table 1. Mothers had a mean age of 26.7 years, 57% had an education level higher than high school, 64.1% had a pre-pregnancy BMI in the overweight or obesity category, and 32.9% had adequate pregnancy weight gain. Mean age of infants was 0.7 months and 52.8% were boys.

Table 2 shows type of feeding in the sample. From a total of 368 infants, only 10 consumed complementary foods, which was distributed as follows: two infants consumed breastmilk and complementary foods, five infants consumed breastmilk, formula, and complementary foods, and three infants consumed formula and complementary foods. Therefore, the data from these 10 infants were removed in subsequent analyses due to the low number of infants in each category. Among those that did not consume complementary foods (n = 358), 39.9% were exclusively breastmilk fed, 47.2% were fed a mix of breastmilk and infant formula, and 12.8% were exclusively formula fed. This distribution was similar by infant sex and maternal pre-pregnancy BMI categories.

Table 3 shows the infant energy and macronutrient intake overall, by sex, and by maternal pre-pregnancy BMI categories. There were no significant differences in the mean intakes of energy, protein, carbohydrates, and fat by infant sex or by maternal pre-pregnancy BMI categories. Also, all infants met energy requirements.

Table 4 shows infant energy and macronutrient intake by feeding type. Intake of energy, protein, fat, and carbohydrates was significantly lower in infants fed only breastmilk compared to infants fed a combination of breastmilk and infant formula and those fed infant formula, after adjusting for important confounders. Also, intake of energy, protein and carbohydrates were lower in infants fed a combination of breastmilk and infant formula compared to infants fed only infant formula, after adjusting for important confounders.

## 4. Discussion

The present study compared energy and macronutrient intake by feeding type and by other infant and maternal characteristics in a sample of 368 infants in Puerto Rico early in life. Our findings revealed that infants fed breastmilk had significantly lower intakes of energy, protein, fat, and carbohydrates than those fed a combination of breastmilk and formula and to those exclusively formula-fed. In addition, those fed a combination of breastmilk and formula also had lower intakes of energy, protein, and carbohydrates compared to those exclusively formula-fed.

There are a few comparative intake studies early in life. A study among 76 healthy, full-term infants from Texas also found lower intake of energy and macronutrients among breastfed infants compared to formula-fed infants at 3 months of age and at 6 months of age but not at 12 or 24 months of age [8]. Another study among 46 healthy, full-term infants from the Netherlands found that infants breastfed had significantly lower intakes of energy, protein, and fat at 1 month, 2 months, and at 4 months but not at 8 or 12 months [7]. No differences were observed for carbohydrate intake at any time point, but sex differences were noted for protein and carbohydrate intake at 1 month only in the study from the Netherlands. Another study among infants in the US also found that breastfed infants had lower energy and protein intakes compared to formula-fed infants at 3 and 6 months [6]. Another study among 1191 White infants from south-west England found that at 4 months, infants exclusively breastfed had the lowest mean intake of protein and carbohydrate but the highest mean intake of total fat compared to formula-fed and mixed-fed infants [5]. It is not clear what the implications are for these differences in energy and macronutrient intake among this group of infants exclusively breastfed compared to the other feeding patterns. It is important to note that all infants, irrespectively of the feeding type, met the estimated energy requirements. Other studies have found that infants exclusively fed infant formula have a greater weight gain and/or weight velocity compared to breastfed infants [6–8]. This greater weight or weight velocity could be contributing to obesity in young children. In the future, we will also evaluate if these early feeding practices in this sample are associated with growth trajectories at 6 months and at 12 months.

The strengths of this cross-sectional analysis include the use of a validated infant FFQ to assess diet, the adjustment of confounders in the statistical analysis, and the relatively large number of minority participants included in the analysis. Nevertheless, some limitations need to be acknowledged when interpreting the results. First, the study’s cross-sectional design does not allow us to establish a causal relationship between the type of feeding and energy and macronutrient intake. Second, the study was conducted in a specific geographic location (WIC clinics in Puerto Rico), which may limit the generalizability of the findings to other Hispanic populations. The breastmilk volume was derived from the FITS cohort study [20], a cohort with some sociodemographic characteristics different from the present cohort (about 25% Hispanics and about 35% were participants of the WIC program, although educational level and age was somewhat similar; BMI or gestational weight gain were not provided in FITS). For infant formula, we did not collect specific brands but used an average nutritional value for several formulas included in the NDSR. Finally, the data analyzed in this study were obtained from the baseline visit of a clinical trial, which may have introduced some selection bias.

## 5. Conclusions

In conclusion, among this underserved Hispanic population and participants of the WIC program in Puerto Rico, we found lower intakes of energy and macronutrients among infants exclusively or partially breastfed compared to infants exclusively formula-fed. The long-term health implications of these findings need to be further studied.

## Figures and Tables

**Table 1.** Socio-demographic data of participants in the Baby-Act trial (N = 368).

Variable	Mean ± SD or N (%)
Infant	
Age (months)	0.71 ± 0.94
Gender	
Female	173 (46.9)
Male	195 (52.8)
Hispanic^1^	341 (92.7)
Race	
White	234 (63.4)
Black	72 (19.5)
American Indian	2 (0.5)
Other	14 (3.8)
Mixed race	12 (3.3)
No answer	7 (1.9)
Maternal	
Age (years)	26.7 ± 5.5
Hispanic	341 (92.9)
Race	
White	190 (51.5)
Black	121 (32.8)
Asian	1 (0.3)
American Indian	6 (1.6)
Other	25 (6.8)
Mixed race	1 (3.0)
No answer	13 (3.5)
Educational level	
Elementary (5 years)	6 (1.6)
Middle (8 years)	4 (1.1)
High School (12 years)	147 (40.0)
Technical/associate (13–14 years)	104 (28.3)
College (15–16 years)	106 (28.9)
Number of children	0.7 ± 0.8
Duration of pregnancy (weeks)	38.7 ± 1.2
Currently working	157 (42.5)
Use of prenatal vitamins^2^	350 (94.9)
Pre-pregnancy BMI (kg/m^2^)^3^	29.8 ± 7.9
Healthy weight	68 (34.9)
Overweight	52 (26.7)
Obesity	73 (37.4)

1 Data from 27 participants are missing;

2 Data from 1 participant are missing;

3 Data from 3 participants are missing.

**Table 2.** Type of feeding overall, by sex of the baby, and by maternal pre-pregnancy BMI (n = 358).

Variable	Only Breastmilk		Breastmilk + Infant Formula		Only Infant Formula		*p*-Value^1^
	N	%	N	%	N	%	
Overall	143	39.9%	169	47.2%	46	12.8%	
Infant sex							
Female	69	19.3%	75	20.9%	23	6.40%	0.703
Male	74	20.7%	94	26.3%	23	6.40%	
Maternal pre-pregnancy BMI^2^							
Healthy weight	47	13.2%	55	15.4%	11	3.10%	0.437
Overweight	43	12.1%	42	11.8%	12	3.40%	
Obesity	51	14.3%	72	20.2%	23	6.50%	

1 No significant differences in the type of feeding distribution by sex or maternal pre-pregnancy BMI.

2 Data from three participants are missing.

**Table 3.** Dietary intake overall, by sex, and by maternal pre-pregnancy BMI categories (n = 358).

Dietary Intake	Overall (n = 358)			Infant Sex							Maternal Pre-Pregnancy BMI Categories^1^									
				Girls (n = 167)			Boys (n = 191)			*p*-Value *	Healthy Weight (n = 113)			Overweight (n = 97)			Obesity (n = 146)			*p*-Value *
	Mean		SD	Mean		SD	Mean		SD		Mean		SD	Mean		SD	Mean		SD	
Energy (kcal/d)	586	±	202	585	±	195	587	±	209	0.875	581	±	220	5787	±	172	585	±	205	0.965
Protein (g/d)	11.8	±	6.28	11.8	±	6.29	11.8	±	6.29	0.968	11.4	±	6.64	11.5	±	5.83	11.9	±	6.13	0.798
Carbohydrate (g/d)	60.7	±	22.9	60.6	±	22.3	60.8	±	23.4	0.900	59.9	±	24.6	59.7	±	19.7	60.6	±	22.8	0.939
Fat (g/d)	33.8	±	9.70	33.7	±	9.15	33.8	±	10.2	0.832	33.9	±	10.7	33.5	±	7.57	33.7	±	10.1	0.967

* No significant difference in the intake of energy and macronutrients by sex of the baby or maternal pre-pregnancy BMI.

1 Data from three participants are missing.

**Table 4.** Energy and macronutrient intake by type of feeding, overall and by sex of the baby (n = 358).

Type of Feeding	Only Breastmilk			Breastmilk and Infant Formula			Only Infant Formula		
	Mean ± SD								
**Overall**	**n = 143**			**n = 169**			**n = 46**		
Energy intake	472 ^a^	±	8.68	637 ^b^	±	222	717 ^c^	±	258
Protein intake	7.1 ^a^	±	0.14	13.7 ^b^	±	6.10	18.1 ^c^	±	6.52
Fat intake	29.8 ^a^	±	0.59	35.9 ^b^	±	11.3	37.5 ^b^	±	13.5
Carbohydrate intake	46.7 ^a^	±	0.92	66.6 ^b^	±	24.3	77. 5 ^c^	±	27.9
**Girls**	n = 69			n = 75			n = 23		
Energy intake	474 ^a^		11.6	625 ^b^		212	712 ^c^		242
Protein intake	7.16 ^a^	±	0.18	13.3 ^b^	±	6.06	18.0 ^c^	±	6.12
Fat intake	29.9 ^a^	±	0.73	35.3 ^b^	±	10.5	37.2 ^b^	±	12.7
Carbohydrate intake	46.9 ^a^	±	1.14	65.4 ^b^	±	23.5	77.0 ^c^	±	26.2
**Boys**	n = 74			n = 94			n = 23		
Energy intake	470 ^a^		6.0	646 ^b^		230	722 ^b^		278
Protein intake	7.10 ^a^	±	0.09	13.9 ^b^	±	6.15	18.3 ^c^	±	7.03
Fat intake	29.7 ^a^	±	0.39	36.3 ^b^	±	11.9	37.7 ^b^	±	14.5
Carbohydrate intake	46.5 ^a^	±	0.59	67.6 ^b^	±	25.1	78.0 ^c^	±	30.1

* Values with different letter superscripts are significantly different in the MANCOVA model, after adjusting for age of the mother, race of the mother, education of the mother, number of previous children, gestational length, and pre-pregnancy BMI (*p* < 0.05). Similar results were found in the unadjusted model.

## Data Availability

The datasets generated during and/or analyzed during the current study are available from the corresponding author on reasonable request.
